# A physical map of human Alu repeats cleavage by restriction endonucleases

**DOI:** 10.1186/1471-2164-9-305

**Published:** 2008-06-26

**Authors:** Murat A Abdurashitov, Victor N Tomilov, Valery A Chernukhin, Sergey Kh Degtyarev

**Affiliations:** 1 SibEnzyme, 2/12 Ak. Timakov Str, 630117 Novosibirsk, Russia

## Abstract

**Background:**

Alu repetitive elements are the abundant sequences in human genome. Diversity of DNA sequences of these elements makes difficult the construction of theoretical patterns of Alu repeats cleavage by restriction endonucleases. We have proposed a method of restriction analysis of Alu repeats sequences *in silico*.

**Results:**

Simple software to analyze Alu repeats database has been suggested and Alu repeats digestion patterns for several restriction enzymes' recognition sites have been constructed. Restriction maps of Alu repeats cleavage for corresponding restriction enzymes have been calculated and plotted. Theoretical data have been compared with experimental results on DNA hydrolysis with restriction enzymes, which we obtained earlier.

**Conclusion:**

Alu repeats digestions provide the main contribution to the patterns of human chromosomal DNA cleavage. This corresponds to the experimental data on total human DNA hydrolysis with restriction enzymes.

## Background

The human genome includes a substantial amount of interspersed repetitive DNA (about 45%) which is divided into several groups (LINEs, SINEs, LTR retroposons, DNA transposons and others) [1]. The Alu family of DNA repeats, which belongs to SINE group, is one of the most abundant and well characterized repetitive elements. The total number of annotated Alu sequences in the database of human genome is more than 1 million copies and their fraction in genome is about 10% [1].

Alu repetitive elements were first described about 30 years ago and were named after conserved AluI restriction site (AGCT), which is present in the most of studied members of the family [2,3]. The Alu repeat sequence is about 300 bp in length and probably originated from the 7SL RNA [4]. The details of Alu repeats retroposition in human genome are still unclear and it is difficult to explain Alu repeats abundance in genomic sequence [5].

Based on the primary structure Alu repeats were divided into at least 9 subfamilies [6,7]. The main branches of Alu subfamilies are designated by one of three possible capital letters, which indicate an age of the repeat group ("J" – old, "S" – intermediate and "Y" – young). Additional lowercase letter and occasional numerical symbols define subfamily name. The most representative Alu subfamilies are AluJb, AluJo, AluSq, AluSx, AluSg and AluY [8]. Similarity of Alu nucleotide sequences within each subfamily varies from 80% to 99% [7,9] and a data base of Alu repeats includes the consensus sequences for some subfamilies [10]. However, quite a big difference in DNA structures between all subfamilies makes difficult a presentation of general Alu repeats consensus sequence covering all subfamilies and there is no such a sequence in Alu repeats database [10].

Earlier we have proposed a simple method to carry out the restriction enzymes analysis of mammalian DNA *in silico *based on the known DNA sequences of eukaryotic genomes [11]. This method allows to calculate lengths of all DNA fragments, which are formed after a whole genome digestion at recognition sites of restriction enzyme, and to construct the distribution diagrams of the calculated DNA fragments. These distribution diagrams display distinct peaks of DNA fragments of the definite lengths due to a presence of DNA repeats in eukaryotic genomes. Comparison of the obtained peaks in distribution diagrams and results of rat, mouse and human DNA cleavage by several restriction enzymes has shown a good correspondence of theoretical and experimental data [11-13]. A detailed study of rat DNA cleavage with restriction enzymes has shown a very good coincidence of DNA digestion patterns *in vitro *and *in silico *for more than 25 restriction enzymes [12]. The obtained patterns of chromosomal DNA cleavage exactly correspond to the theoretical patterns of the proposed consensus rat LINE1 repeat cleavage [12]. A similar study of human chromosomal DNA digestion [13] has revealed another situation, where an obtained theoretical pattern of DNA cleavage is formed as a mix of Alu and LINE1 repeats digestion patterns. Most of small DNA fragments are produced from Alu repeats, whereas DNA fragments of length more than 300 bp are formed from LINE1 repeats. In this work we have studied in details Alu repeats digestion *in silico*. We have described an approach to analyze a big family of human Alu repeats from the data base, which comprises of more than 1 million of homologous short nucleotide sequences. The calculated data have been compared with digestion of subfamilies' Alu repeats and with experimental results of human DNA cleavage by restriction endonucleases, which have been obtained earlier [13].

A goal of this work is to 1) develop a software for analysis of a big number of Alu repeats, 2) compare the calculation data on cleavage of the whole human genome and a set of Alu repeats, 3) carry out a comparative analysis of experimental results of DNA hydrolysis and theoretical data on various Alu repeats families' digestion and 4) present a physical map of Alu repeats cleavage with 9 restriction enzymes.

## Results and discussion

### Comparison of distribution diagrams for the whole human genome and the set of Alu repeats

Earlier we have shown that mammalian DNAs hydrolysis with restriction enzymes and subsequent DNA products separation in gel-electrophoresis produce cleavage patterns, which mainly correlate to the DNA fragment peaks in distribution diagrams obtained by computer analysis of published genomes [11-13]. The only exception is α-satellite DNA digestion products, which give clearly visible DNA fragments whereas they have no corresponding peaks in diagrams [11-13]. Unfortunately, the calculation of α-satellite DNA cleavage patterns is impossible because of absence of multisatellite DNAs nucleotide sequences in the informational databases.

The existence of peaks in diagrams is explained by the presence of a large amount of repetitive DNA in eukaryotic genomes. Indeed, we have shown that almost all peaks in diagrams of rat genomic DNA digestion *in silico *correspond to the peaks of rat LINE1 consensus sequence cleavage [12]. The human genome differs from rat genome by a larger proportion of rather short SINE repeats (about 300 bp in length and mainly belonging to Alu family). As a result 30–300 bp DNA fragments in digestion diagrams are formed from Alu repeats and we have provided two distribution diagrams of Alu repeats digestion in our previous work [13]. In this work we have developed software and carried out a detailed analysis of Alu repeats cleavage with restriction endonucleases.

As indicated in "Methods" each nucleotide sequence from more than 1,190,000 annotated Alu repeats sequences has been searched for a presence of recognition sequence of restriction enzymes, a number and positions of these sites in Alu repeat sequences have been determined. Then, Alu repeats digestion diagram, a diagram of the obtained DNA fragments number versus the length of these fragments has been plotted for each recognition sequence.

**Figure 1:**
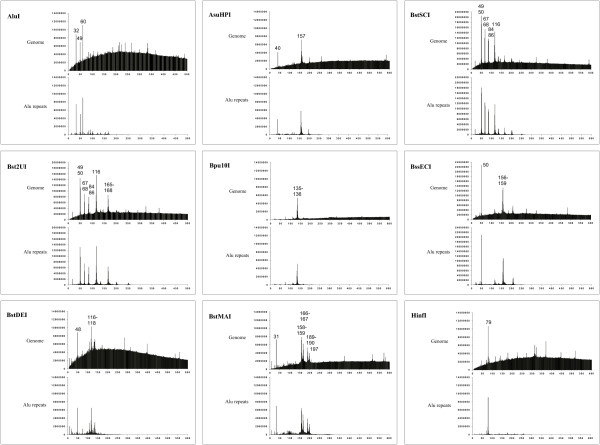
**Comparison of distribution diagrams**. Distribution diagrams obtained for complete human genomic sequence are shown at top, whereas those for the complete set of annotated Alu repeats are shown at bottom. The highest peak values are indicated.

Earlier we have observed a similarity between distribution diagrams of whole genome and Alu repeats digestion in the case of AluI and Bst2UI recognition sequences [13]. Fig. 1 shows a comparison of distribution diagrams of a whole genome and Alu repeats digestion for recognition sites of 9 restriction enzymes. As it is clearly seen from the figure all peaks of 300 bp and less are present both in diagrams of the whole human genome digestion and in diagrams of the annotated Alu repeats cleavage *in silico*. Thus, indeed, the corresponding fragments of genomic DNA are formed due to a cleavage of Alu repeats. A cleavage of remaining DNA forms a background curve of a whole genomic DNA digestion. A structure of this background fragments curve depends mainly on GC-content of genomic DNA, enzymes recognition site and has been discussed earlier [12]. On Fig. 1 there are some smaller peaks in whole genome cleavage diagrams. Our analysis has shown that these peaks are formed mainly due to a cleavage of human LINE1 repeat (data not shown).

### Alignment and analysis of Alu repeats

**Figure 2:**
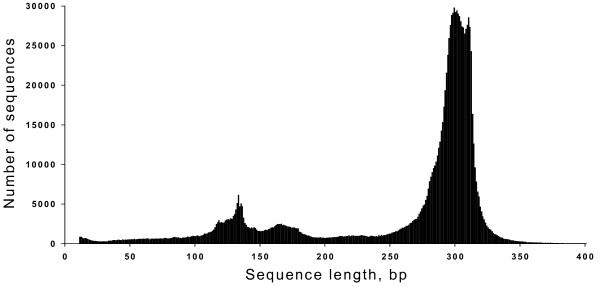
Distribution of Alu repeats sequences depending on their lengths.

Preliminary analysis has shown that Alu repeat sequences differ in length. On Fig. 2 we have presented a diagram of Alu repeats number versus a length of Alu repeats sequences, which are presented in data base. Fig. 2 shows that a big number of Alu repeats shorter than 250 bp are listed in data base. We have made some additions to 5'-truncated short Alu repeats sequences to unify them for a subsequent analysis. As indicated in "Methods" we have aligned all DNA sequences, which originally were 5'-truncated, by adding extra nucleotides at 5'-end of Alu repeats. A total number of all obtained human Alu repeat sequences, which has been considered in a further analysis, is 1,193,407. These aligned Alu repeats are presented at SibEnzyme data base [14].

**Figure 3:**
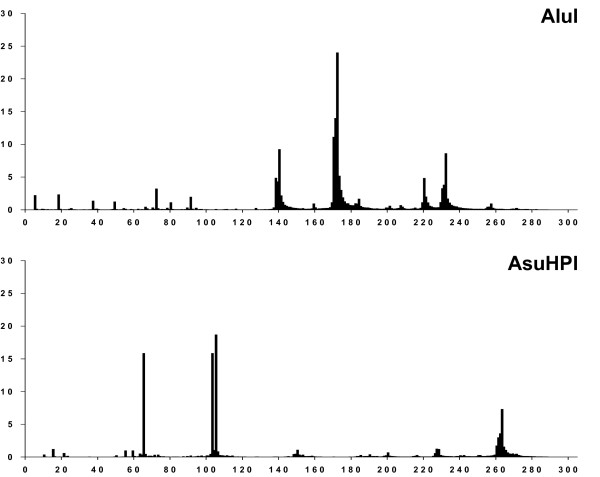
Distribution diagrams of AluI and AsuHPI recognition sites in all Alu repeats sequences.

At the first step we have plotted a distribution diagram of restriction enzymes recognition sites at every position of Alu repeat for all aligned Alu repeat sequences. Fig. 3 shows these distribution diagrams for restriction enzymes AluI and AsuHPI. As we can see from Fig. 3 there are distinct peaks of RE sites in Alu repeats sequences for AluI and AsuHPI restriction endonucleases as well as for all other enzymes, which we have studied here (see Additional file 1: Distribution diagrams of restriction enzymes' recognition sites in all Alu repeats sequences). For a further analysis locations of the maximal peak's value were chosen as these sites positions. It should be noted that some peaks are very close each to other (a twin peak) due to a common 2 bp deletion in positions 65–66 [6]. We have observed twin peaks in positions 99/101 (AsuHPI), 79/81 and 110/112 (BstMAI), 87/89 (Bst2UI). Table 1 shows positions of recognition sites in aligned Alu repeats sequences and a number of sites at these positions in all Alu repeats (a combined value for a number of sites in twin peaks is given in the table). Because of short deletions and/or insertions in many Alu repeats sequences we have applied an approach of each site number determination as follows. Every particular recognition sequence has been searched in the interval ± 5 bp of the position indicated in column 2. In column 3 of Table 1 we have presented a total number of these sites. For the further consideration we have chosen only those sites, which are present at every particular position in more than 100,000 Alu repeats sequences. In Table 1 we have also indicated the percentage of Alu repeats, which contain a given site, and this site distribution within main subfamilies (in %).

**Table 1:** Amount of Alu repeats, which contain restriction enzymes recognition site at the certain positions

**Restriction enzyme (Recognition site)**	Position of site*	Number of Alu repeats with recognition site*	**% of Alu repeats with a RE site**	**% of Alu repeats with RE site in the main subfamilies**					
				
				**AluJb **(129,921 copies)	**AluJo **(143,179 copies)	**AluSq **(95,474 copies)	**AluSx **(342,315 copies)	**AluSg **(82,849 copies)	**AluY **(139,479 copies)
**AluI **(AGCT)	136	284,829	24	22	20	31	28	32	18
	**168**	**751,006**	63	53	47	76	72	78	78
	216	134,112	11	6	8	2	3	2	65
	228	261,288	22	20	33	22	23	22	19

**AsuHPI **(GGTGA)	61	213,081	18	2	1	54	33	1	1
	**99/101**	**453,607**	38	18	2	58	51	63	64
	259	243,227	20	23	18	11	27	28	20

**Bpu10I **(CCTNAGC)	46	229,143	19	18	19	21	22	22	16
	**181**	**633,313**	53	41	35	63	59	68	72

**BssECI **(CCNNGG)	**47**	**480,634**	40	27	24	48	47	50	59
	89	190,713	16	47	46	3	6	2	2
	**205**	**639,461**	54	44	38	62	59	65	71
	**255**	**607,377**	51	42	35	62	59	64	72

**BstDEI **(CTNAG)	47	273,350	23	23	25	24	26	25	18
	65	281,670	24	16	8	60	41	1	1
	173**	276,082	23	24	23	25	26	36	20
	182**	**760,759**	64	55	49	75	72	78	79
	230	184,220	15	9	3	22	23	22	14

**BstMAI **(GTCTC)	**79/81**	**513,948**	43	31	29	49	47	52	66
	**110/112**	**461,551**	39	27	26	44	42	49	60
	270***	**391,589**	33	31	23	10	41	44	53
	278***	**410,275**	34	36	29	31	37	36	50

**HinfI **(GANTC)	193	344,902	29	6	4	53	46	50	5
	**272**	**422,725**	35	7	7	10	53	63	71

**Bst2UI **(CCWGG)	3	257,322	22	20	19	25	26	25	20
	71	183,209	15	37	43	3	10	2	2
	**87/89**	**658,863**	55	49	48	62	66	58	76
	138	270,331	23	23	21	27	28	27	23
	**205**	**494,290**	41	41	37	48	48	48	38
	**255**	**640,134**	54	42	36	62	60	65	72

Data are given for all analyzed sequences and for six main subfamilies.

* – Positions and numbers of sites which present in more than 30% of Alu repeats are shown in bold.

** – Because of possible overlapping the following regions were considered: 168–177 and 178–187.

*** – Because of possible overlapping the following regions were considered: 265–274 and 275–283.

According to Table 1 data a maximal number of restriction enzymes' recognition sites in one position is 750,000 – 760,000. We have got such number of AluI sites at position 168 and BstDEI sites at position 182. Another set of sites in a range of 605,000 – 660,000 includes Bpu10I recognition sequence at position 181, two sites of BssECI at positions 205 and 255 and two sites of Bst2UI at positions 87/89 and 255. So, 63–64% of all aligned Alu repeats sequences contain AluI site at position 168 and BstDEI site at position 182. This value corresponds to a general estimation of AluI sites presence in the set of Alu repeats [2].

At the second step we have calculated a number of all DNA fragments produced in the course of Alu repeats digestion at the recognition sites of the enzymes indicated in Table 1. In "Methods" we have described a special approach to take into account the heterogeneity of Alu repeats because of short deletions and insertions. In calculations of DNA fragments length we have considered an accuracy ± 2 bp for DNA fragments less than 100 bp, ± 3 bp for DNA fragments 100–200 bp and ± 4 bp for DNA fragments more than 200 bp. Due to clustering effect in experiment [12] the intensities of the bands, which correspond to DNA fragments of similar sizes, are summarized on the gel pictures. The bigger fragments are resolved in gel-electrophoresis less effective than smaller fragments. Thus, such an approximation allows us to take into account Alu repeats with possible small deletions or insertions. In Table 2 we have summarized the obtained data. For each fragment a total number of bp in corresponding peak in diagram has been determined (column 5). Earlier we have shown [11,12] that visualization of DNA fragments in gel electrophoresis experiments is possible if a total number of bp in peaks in DNA fragments distribution diagrams is about 4,5 million bp and higher. DNA fragments of such size are selected in column 6 of Table 2.

**Table 2:** Length and number of DNA fragments produced by cleavage of Alu repeats

**Enzyme**	Fragment length*	Positions*	**Number of sequences, thousands**	**Total bp number in DNA fragment, millions bp**	**Total bp number in selected DNA fragment(s), millions bp**
**AluI**	92 ± 2	136–228	9,9	0,9	
	80 ± 2	136–216	2,3	0,2	
	**60 ± 2**	**168–228**	**151,8**	**9,1**	**9,1**
	**48 ± 2**	**168–216**	**98,7**	**4,8**	**4,8**
	**32 ± 2**	**136–168**	**221,8**	**7,1**	**7,1**
	12 ± 2	216–228	20,3	0,2	

**AsuHPI**	198 ± 3	61–259	13,6	2,7	
	**159 ± 3**	**100–259**	**86,8**	**13,7**	**13,7**
	39 ± 2	61–100	97,0	3,9	

**Bpu10I**	**135 ± 3**	**46–181**	**107,7**	**14,5**	**14,5**

**Bst2UI**	252 ± 4	3–255	10,4	2,6	
	202 ± 4	3–205	18,1	3,7	
	184 ± 3	71–255	4,4	0,8	
	**167 ± 3**	**88–255**	**102,0**	**17,0**	**17,0**
	135 ± 3 134 ± 3	3–138 71–205	15,5 10,3	2,1 1,4	
	**117 ± 3 117 ± 3**	**88–205 138–255**	**172,2 66,8**	**20,1 7,8**	**27,9**
	**85 ± 2**	**3–88**	**123,7**	**10,5**	**10,5**
	68 ± 2	3–71	41,0	2,8	
	**67 ± 2**	**138–205**	**125,1**	**8,4**	**11,8**
	67 ± 2	71–138	9,3	0,6	
	**50 ± 2**	**88–138**	**139,5**	**6,9**	**22,9**
	**50 ± 2**	**205–255**	**323,7**	**16,0**	
	17 ± 2	71–88	110,0	2,0	

**BssECI**	**208 ± 4**	**47–255**	**47,8**	**9,9**	**9,9**
	166 ± 3	89–255	15,8	2,6	
	**158 ± 3**	**47–205**	**217,6**	**34,3**	**34,3**
	**116 ± 3**	**89–205**	**53,5**	**6,2**	**6,2**
	**50 ± 2**	**205–255**	**405,6**	**20,3**	**20,3**
	42 ± 2	47–89	41,5	1,7	

**BstDEI**	183 ± 3	47–230	2,3	0,4	
	165 ± 3	65–230	4,7	0,8	
	**135 ± 3**	**47–182**	**68,1**	**9,2**	**9,2**
	126 ± 3	47–173	34,6	4,3	
	**117 ± 3**	**65–182**	**114,1**	**13,3**	**13,3**
	**108 ± 3**	**65–173**	**53,8**	**5,8**	**5,8**
	57 ± 2	173–230	6,7	0,4	
	**48 ± 2**	**182–230**	**133,7**	**6,4**	**6,4**
	18 ± 2	47–65	65,4	1,2	

**BstMAI**	**198 ± 3**	**80–278**	**38,6**	**7,6**	**7,6**
	**190 ± 3**	**80–270**	**58,7**	**11,1**	**11,1**
	**167 ± 3**	**111–278**	**78,9**	**13,1**	**13,1**
	**159 ± 3**	**111–270**	**125,4**	**19,9**	**19,9**
	**31 ± 2**	**80–111**	**235,0**	**7,3**	**7,3**
	8 ± 1	270–278	167,6	1,3	

**HinfI**	**79 ± 2**	**193–272**	**144,4**	**11,4**	**11,4**

Data for DNA fragments with total bp numbers exceeding 4.5 mln bp are shown in bold.

* If there are two positions of the site, an average value is indicated (for example, 99/101 positions for AsuHPI site are replaced with 100).

### Comparison of theoretical and experimental data

**Figure 4:**
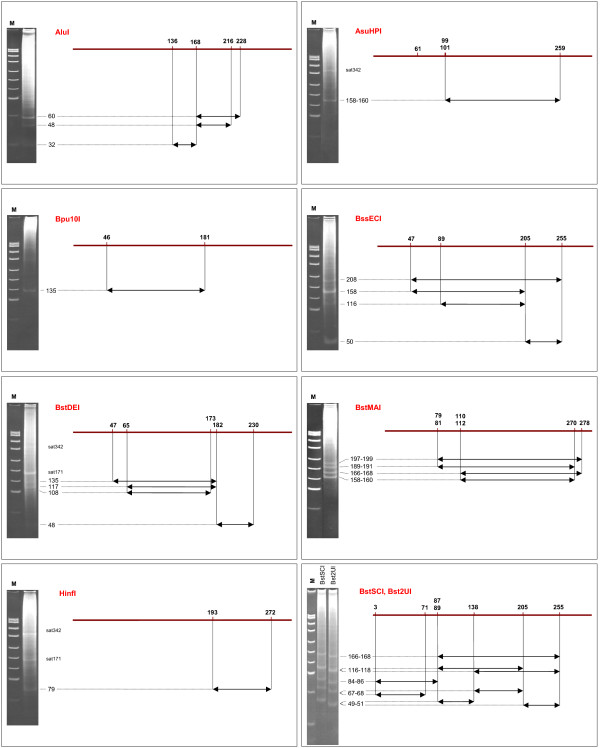
**Restriction maps of Alu repeats**. Most conserved recognition sites of restriction enzymes are shown at top. Electrophoregrams of human genomic DNA enzymatic cleavage (8% PAAG) and lengths of fragments are shown at left. A correspondence of predicted DNA fragments (arrows) to the bands in electrophoregrams is shown by dotted lines. M – DNA ladder pUC19/MspI (the lengths of visible fragments are: 501, 489, 404, 331, 242, 190, 147, 111+110, 67 and 34 bp, from top to bottom). "sat" – α-satellite DNA cleavage products.

On Fig. 4 we provide a physical map of Alu repeats cleavage by various restriction endonucleases. This map has been constructed based on the data given in Table 2. Experimental data on chromosomal DNA cleavage [13] are given on a left side of Fig. 4. There is a good correspondence of the experimental results and the theoretical data, which is discussed in details below.

Alu repeats digestion at recognition sequence AGCT produces 3 DNA fragment: 60 bp, which is produced by cleavage at positions 168 and 228, 49 bp – at positions 168 and 216 and a small fragment 32 bp – at positions 136 and 168. So, we have a common site 168 for all obtained DNA fragments and a different location of the second AluI site. Surprisingly, AluI site at position 216 is present mostly in AluY subfamily. AluY subfamily contains 139,479 sequences and this value is higher than 98,7 thousand of 49 bp fragments given in Table 2. This difference is, probably, because a great part of AluY sequences have only one AluI site (either at 168 or at 216).

Restriction enzyme AsuHPI has three sites on Alu repeats at 61, 99/101 and 259. However, human DNA cleavage with AsuHPI produces only one band, which corresponds to 158/160 bp DNA fragment (Alu repeats digestion at 99/101 – 259) and there is 342 bp fragment of satellite DNA cleavage [15]. AsuHPI site at position 61 is present mostly in AluSx and AluSq subfamilies and the quantities of 61–168 and 61–259 DNA fragments are less than 4,5 million bp and are not visible in gel-electrophoresis experiments [12]. It's interesting to note that AsuHPI site at position 99/101 occurs in AluJo subfamily in minimal quantities (2%).

Bpu10I cleaves human DNA with formation of one visible band, which corresponds to 135 bp DNA fragments (46–181 positions in Alu repeats).

As we showed earlier in our calculations [13] a human chromosomal DNA digestion with BssECI produced more than 400 thousand of 50 bp DNA fragment and this is the highest fragments number among all studied distribution diagrams. As indicated in Table 2 a number of 50 bp DNA fragments in Alu repeats digestion at site CCNNGG is 405.6 thousand and this value closely correlates to the data on a whole genome cleavage. In accordance with Table 2 we see good bands, which correspond to 50 bp (positions 205–255) and 158 bp (positions 47–205) DNA fragments and a couple of weak bands corresponding to 116 bp (positions 89–205) and 208 bp (positions 47–255) DNA fragments. So, experimental and theoretical data on Alu repeats digestion with BssECI show a good correlation. The position 89 occurs mainly in "old" AluJo and AluJb repeats.

Distribution of BstDEI sites in Alu repeats is similar to distribution of AluI sites: there is a main BstDEI cleavage position at 182, which is a part of Bpu10I site at 181, and smaller portions of Alu repeats have BstDEI sites at positions 47, 65, 173 and 230. Surprisingly, more than 270,000 Alu repeats contain DNA sequence CTNAG (recognition site of restriction enzyme BstDEI) at position 47, whereas more than 480,000 Alu repeats contain site CCNNGG (recognition sequence of enzyme BssECI) at the same position. These data show a presence of T and C in position 48 of more than 270 thousand and more than 480 thousand of Alu repeat sequences, respectively. As a result we observe weak bands 48, 108, 117 and 135 bp, which correspond to DNA fragments 182–230, 65–173, 65–182 and 47–182, and 171 bp fragment of satellite DNA cleavage [15]. It should be noted that AluSg and AluY subfamilies practically have no BstDEI site at position 65.

Distribution of all BstMAI sites on Alu repeats is more uniform and these sites are located at positions 79/81, 110/112, 270 and 278 in all subfamilies. In accordance with a Table 2 data and a map of Alu repeats cleavage (Fig. 4) we see all possible DNA fragments (79/81–270, 79/81–278, 110/112–270 and 110/112–278) on the gel photo.

Alu repeats cleavage with HinfI produced one DNA fragment 79 bp (positions 193–272), which may be observed on the gel photo along with 171 and 342 bp DNA fragments, which are the products of satellite DNA cleavage. Analysis of HinfI site distribution has shown that GANTC sequence at position 193 is presented mostly in AluSq, AluSx and AluSg, whereas GANTC sequence at 272 occurs mainly in AluSx, AluSg and AluY subfamilies.

Digestion of Alu repeats with Bst2UI and BstSCI mostly takes place at six positions: 3, 71, 87/89, 138, 205 and 255. Due to a high level of methylated CG sites in human genome BstSCI cleaves mainly CCWGG sites, which are Bst2UI recognition sequence. Thus, both enzymes have the same patterns of DNA cleavage and the same band intensities on the gel photo. A shift of BstSCI DNA cleavage pattern was discussed earlier [13] and in a further work we have studied a map of Bst2UI cleavage only. Three Bst2UI recognition sites (87/89, 205 and 255) coincide with locations of BstECI sites in Alu repeats. Sites at positions 87/89, 205 and 255 are presented in the most of Alu repeats and we can see the same DNA fragments 50 bp (positions 205–255), 115–117 bp (positions 87/89–205) and 166–168 bp (positions 87/89–255) for DNA cleavage with enzymes BstSCI, Bst2UI and BstECI on gel photos. Quite a large portion of Alu repeats have Bst2UI site at position 3. A corresponding DNA fragment 84/86 bp (positions 3–87/89) we can see on the gel photo. Some Alu repeats have additional Bst2UI sites at positions 71 and 138. In accordance with a map of Alu repeats digestion on the photo we see the additional bands, which correspond to DNA fragments 68 bp (positions 3– 71), 49 bp (positions 89–138, this band is overlapping with 50 bp band) and 67 bp (positions 138–205). So, Alu repeats digestion with Bst2UI provides three couples of overlapping DNA fragments: 49 bp and 50 bp, 67 bp and 68 bp, 115 and 119 bp. A good picture of the gel photo with bright bands of human DNA hydrolysis with BstSCI and Bst2UI enzymes [13 and data of this article] may be explained by presence of Alu repeats digestion fragments in doubles. AluSq, AluSg and AluY subfamilies contain minimal quantities of Bst2UI site at position 71.

## Conclusion

A method of theoretical analysis of Alu repeats sequences from the database has been proposed in this work. It includes a) a search for restriction enzymes recognition sites in all Au repeats sequences, b) additional alignment of annotated Alu repeats sequences with formation a new set of Alu repeats sequences, c) determination of location of the restriction enzymes recognition sites in a new set of Alu repeats and calculation of a number of these sites in each positions, d) calculation of a number of DNA fragments, which are produced in the course of Alu repeats cleavage at these sites. We also have compared data on sites' distribution among main Alu repeats subfamilies. There is a definite preference in distribution of some restriction enzymes recognition sequences among different families of Alu repeats, for example, AluI site at position 216 is characteristic for "young" AluY sequences. Thus, such preference may be used to characterize Alu subfamilies by restriction enzymes analysis. Finally, we have constructed a physical map of Alu repeats cleavage by restriction endonucleases and have shown a good correspondence of theoretical and experimental data.

The suggested method of Alu repeats analysis allows to simplify a study of human DNA digestion with restriction endonucleases considering set of Alu repeats sequences instead of the whole human genomes. Thus, time and efforts, which are necessary for such calculations, may be significantly reduced.

## Methods

### Nucleotide sequence data

The human genomic DNA sequence was obtained from Ensembl project FTP (version of November 28, 2006) [16]. The complete set of annotated Alu repetitive elements was obtained from the UCSC Genome Browser website using Table Browser service, human genomic assembly hg18 (version of March 2006), "Variation and repeats" group and family name ("Alu") as filter value in "repFamily" field [17]. The set includes 1,193,407 sequences with a total length of ~350 million bp.

Primary analysis has shown that many of these sequences were 5'-truncated. So, for a further analysis and mapping these truncated sequences were aligned as follows. Six consensuses of most abundant Alu subfamilies (AluJb, AluJo, AluSq, AluSx, AluSg and AluY) were used as reference sequences. Each Alu repeat sequence from the complete set was compared to the each consensus sequence starting from different positions. For each Alu repeat sequence a maximal number of coinciding nucleotides with the analyzed Alu repeat subfamily's consensus has been determined and starting position of this sequence has been calculated referring the given consensus sequence. Additional symbols "N" in number, which is equal a starting position – 1 nucleotide, have been added to the 5'-end of the analyzed Alu repeat sequence.

### Sequence analysis and diagram plotting

The DNA fragments distribution diagrams for a whole human genome digestion at the recognition sites of restriction endonucleases have been obtained in our previous publication [13]. The distribution diagrams for Alu repeats digestion at the recognition sites of restriction endonucleases AluI and Bst2UI have been presented earlier [13]. The DNA fragments distribution diagrams of Alu repeats digestion at the recognition sites of restriction endonucleases AsuHPI, BstSCI, Bpu10I, BssECI, BstDEI, BstMAI and HinfI were constructed according to the previously described technique [11] with some modifications as described below.

The calculation algorithm includes the following steps: i) to load a single Alu sequence from the set of annotated Alu repeat sequences, ii) to perform an optimized search for a definite recognition site within selected Alu repeat sequence; iii) to calculate the distances between nearest recognition sites (lengths of fragments), which have been found. These three steps were repeated for every sequence in the database of annotated Alu repeats sequences. A total number of fragments of each size was summarized for all possible lengths of DNA fragments. The data obtained were exported to a file in CSV (comma separated values) format, where the first number represented the fragment length, the second – a number of the fragments of the indicated length in all Alu set, and the third – a total bp number in all fragments of the same length (column 1 value multiplied by column 2 value). These data were imported to Microsoft Excel table and used to plot diagrams.

### Determination of DNA fragments which may be visible on gels

A simple computer program has been developed for calculation of number of the sequences, which contain the same recognition sites at two positions corresponding to the peaks in diagrams on Fig. 3 or in the Additional file 1. A complete algorithm includes following steps: 1) the search for a definite recognition site at the first selected region (first peak position ± 5 bp); 2) determination of position for the recognition site next to the first one; 3) calculation of the distance (fragment length) between these neighbouring sites; 4) verification of the distance obtained to the predicted length of DNA fragment in the column 2 of the Table 2 (with accuracy ± 2 bp for fragments less than 100 bp in length, ± 3 for fragments of 100–200 bp in length and ± 4 for fragments more than 200 bp in length); 5) positive results of DNA fragments comparison have been summarized and presented in the column 4 of the Table 2. The summarized quantities of base pairs in all DNA fragments of the indicated length have been calculated and presented in the column 5 of the Table 2.

### Alu repeats restriction maps construction

For each restriction endonuclease the data on recognition site positions in the set of Alu repeats have been extracted from Table 1. The data on valuable number of each DNA fragment have been obtained from the Table 2.

The photographies of gels after electrophoresis of human DNA products digestion with restriction endonucleases have been obtained in our previous publications [11,13].

## Authors' contributions

MAA carried out the data analysis and drafted the manuscript. VNT developed software and participated in computer analysis. VAC carried out a comparison of the obtained results with experimental data on DNA hydrolysis. SKD coordinated the project and prepared the final manuscript. All authors have read and approved the final manuscript.

## Supplementary Material

Additional file 1 Distribution diagrams of restriction enzymes' recognition sites in all Alu repeats sequences. Click here for file
