# Supplementary figures and images for: A physical map of human Alu repeats cleavage by restriction endonucleases
Source: BMC Genomics. 2008 Jun 26;9:305. doi: 10.1186/1471-2164-9-305 (PMC2443384; doi:10.1186/1471-2164-9-305)

**AluI**

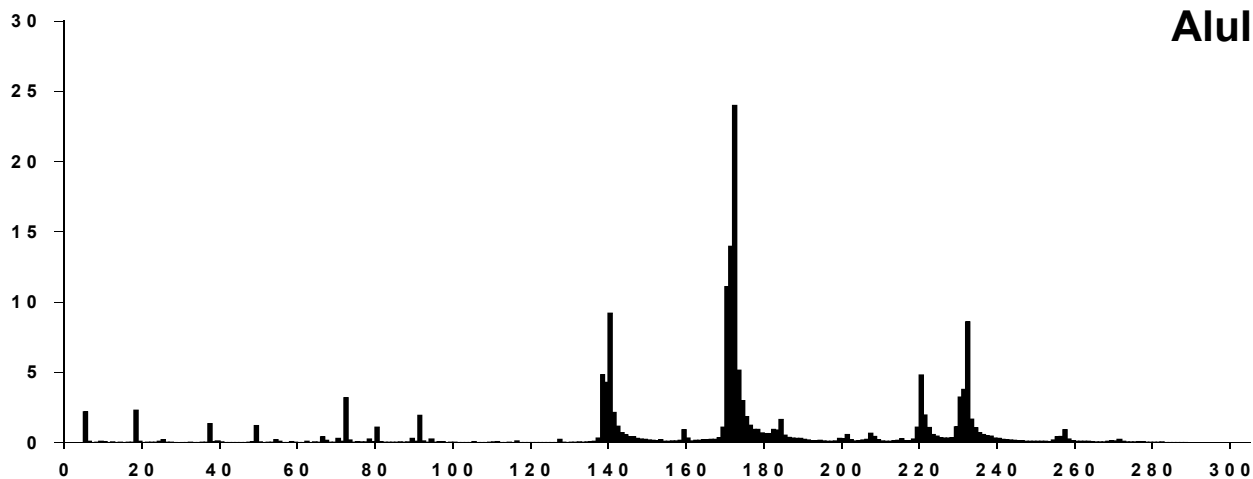

**AsuHPI**

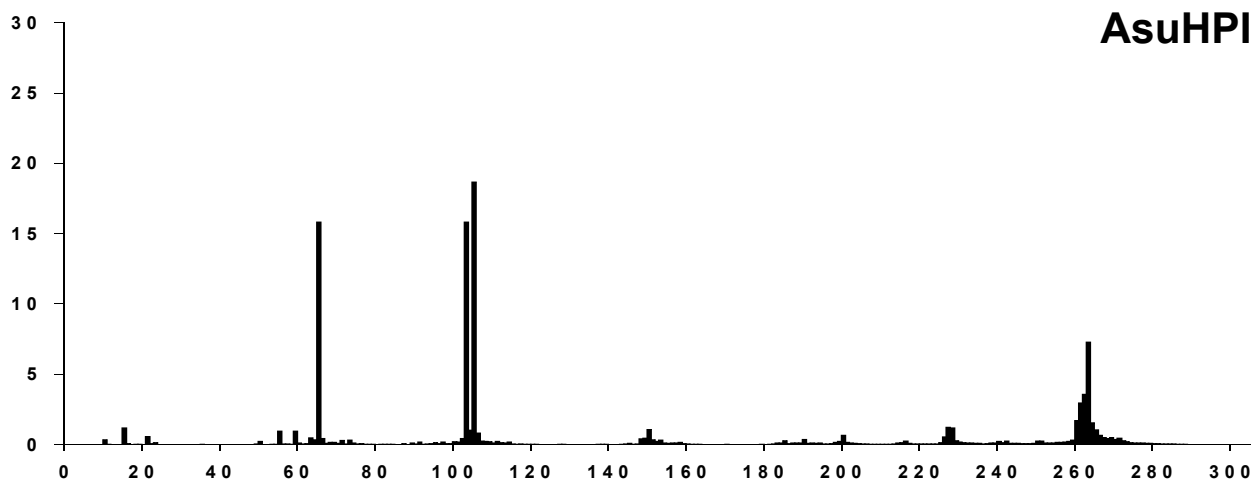

**Bpu10I**

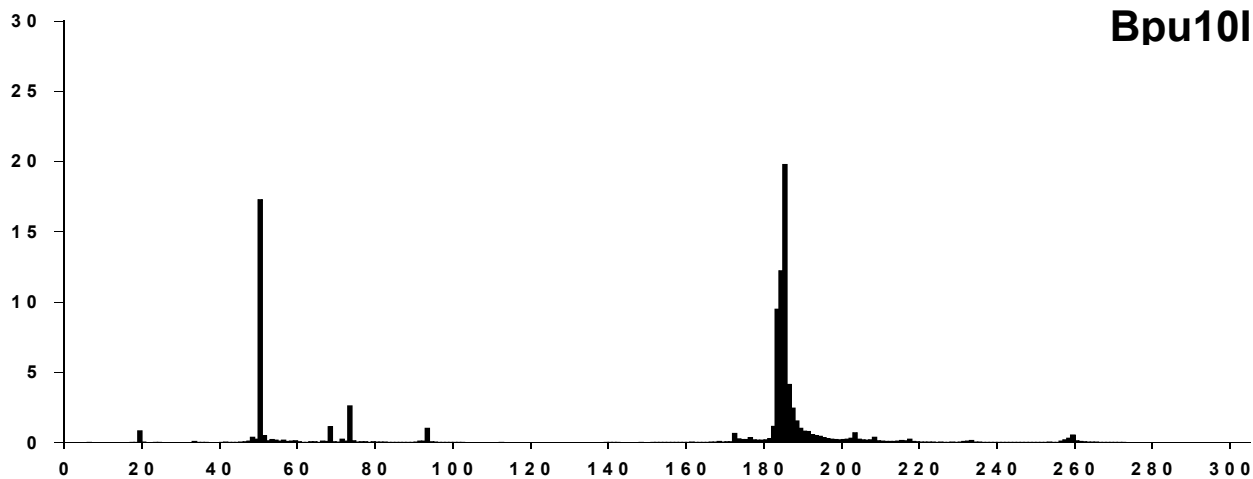

**BssECI**

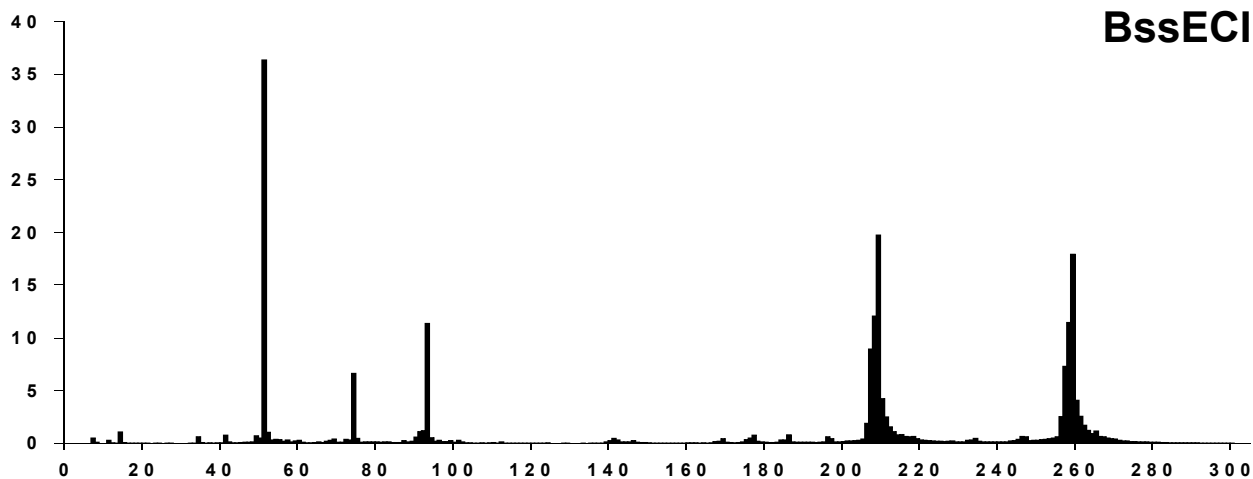

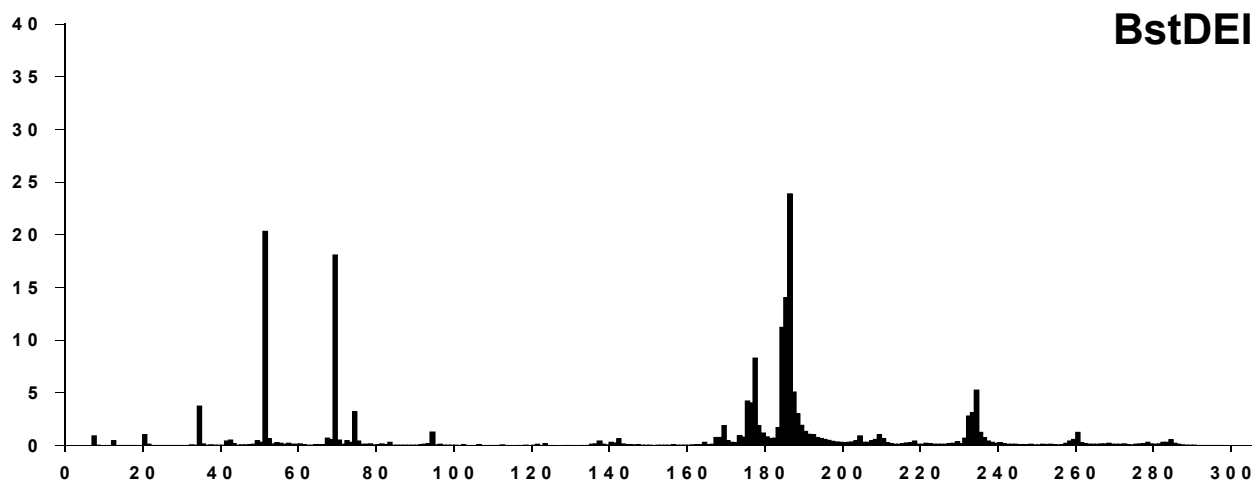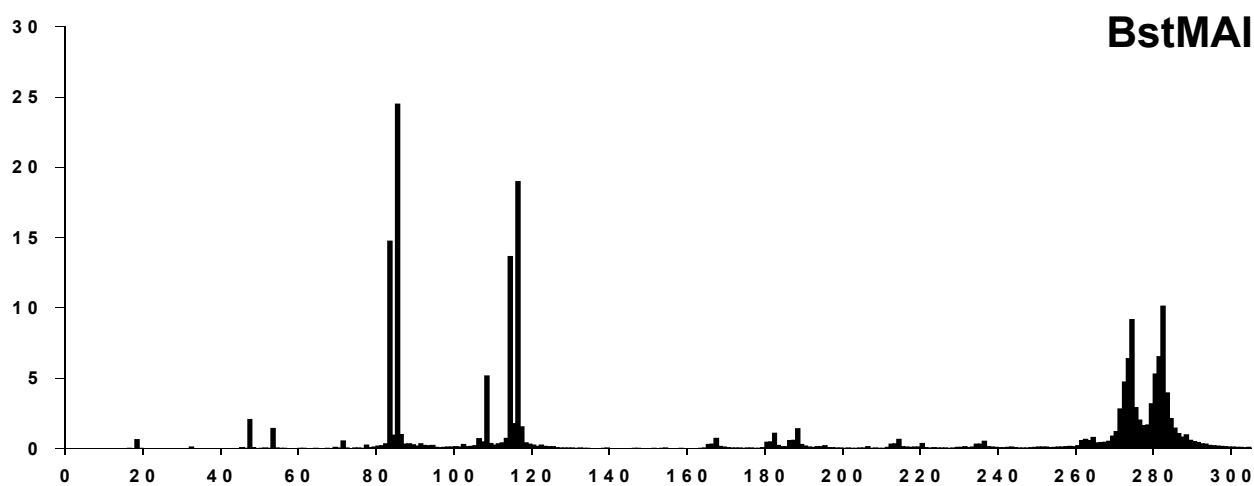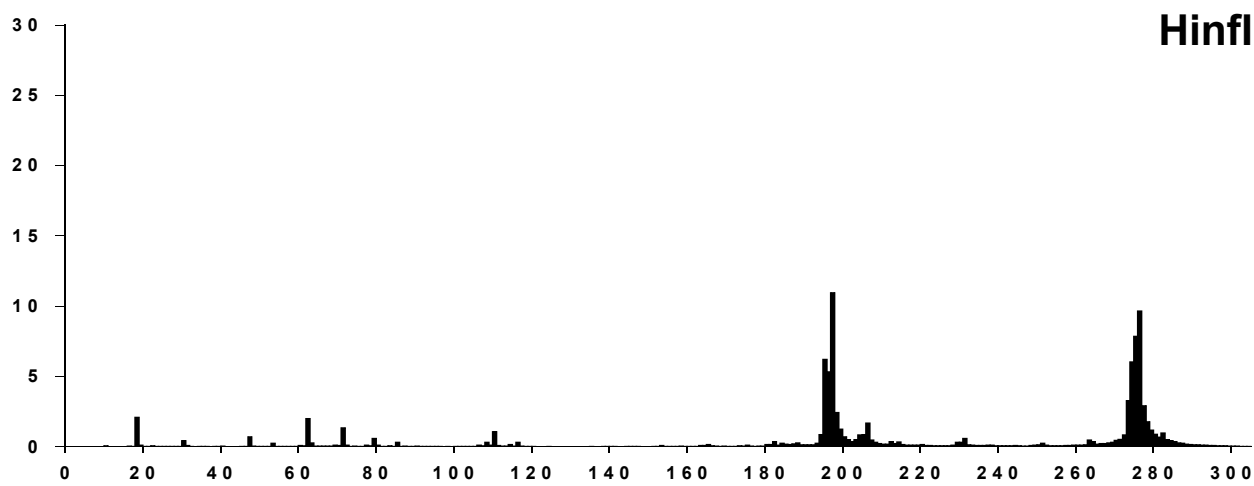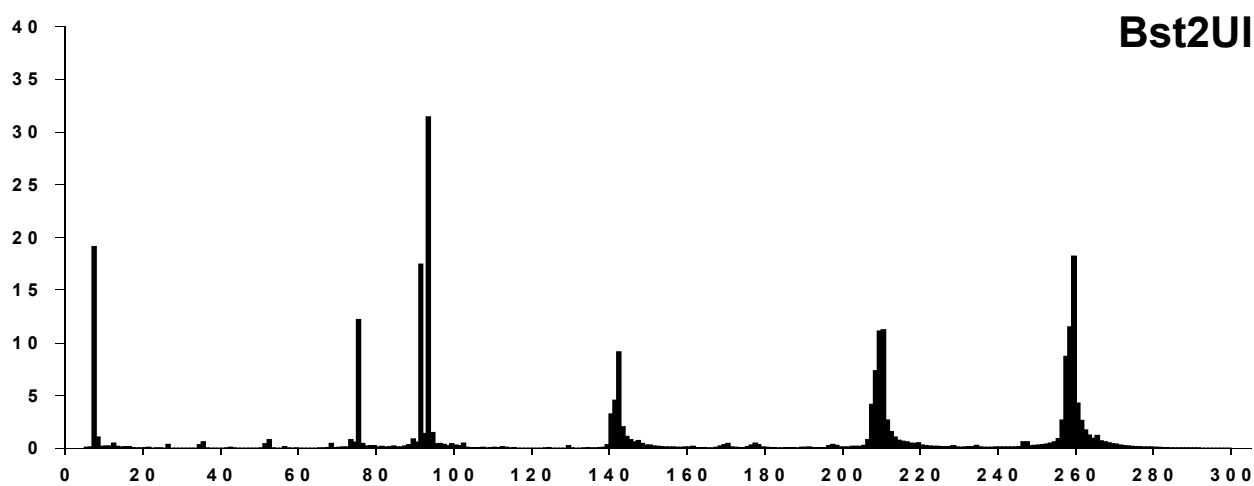

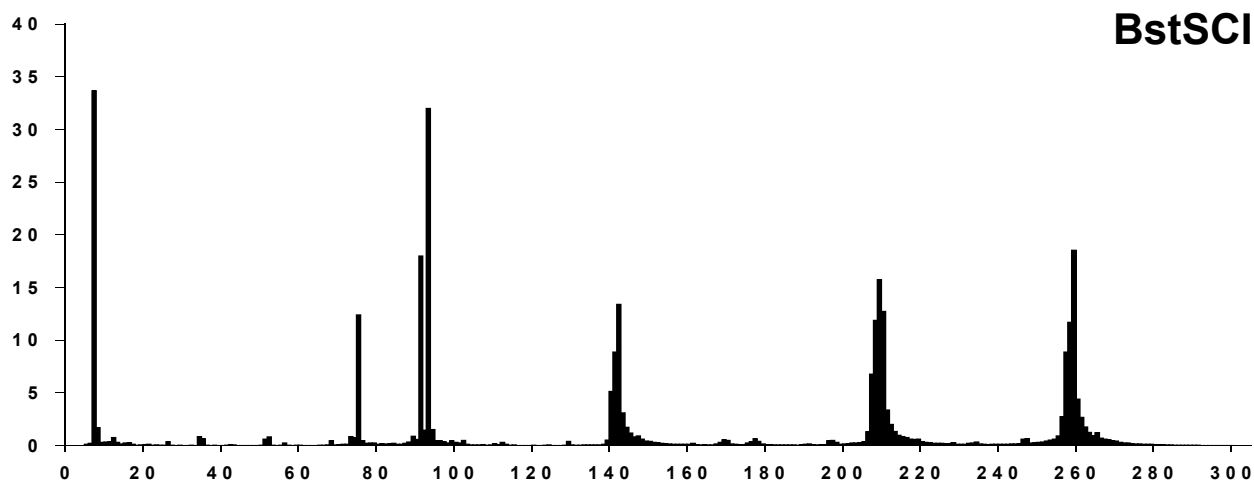

Supplement: Additional file 1 — Distribution diagrams of restriction enzymes' recognition sites in all Alu repeats sequences. [file 1471-2164-9-305-S1.pdf]
